# Molecular Mechanisms Linking Obesity and Type 2 Diabetes: Pathogenesis, Biomarkers, and Therapeutic Potential

**DOI:** 10.3390/cimb48101079

**Published:** 2026-10-19

**Authors:** Jiwon Han, Apeksha Kamat, Jasmin Nguyen, Phat Nguyen, Katelyn Vu, Melissa Zhao, Vishwanath Venketaraman

**Affiliations:** College of Osteopathic Medicine of the Pacific, Western University of Health Sciences, Pomona, CA 91766, USA; jiwon.han@westernu.edu (J.H.); apeksha.kamat@westernu.edu (A.K.); jasmin.nguyen@westernu.edu (J.N.); phat.nguyen@westernu.edu (P.N.); katelyn.vu@westernu.edu (K.V.); melissa.zhao@westernu.edu (M.Z.)

**Keywords:** obesity, diabetes, inflammation, biomarkers

## Abstract

Obesity and diabetes are major global health burdens, with their prevalence projected to continue rising. The comorbidity of obesity and diabetes increases the risk of serious complications and mortality, highlighting the need for early prevention and intervention. However, the precise mechanisms underlying their relationship remain unclear. A review of ten articles selected from PubMed and published from 2016 to 2026 was conducted to study the pathogenesis of type 2 diabetes (T2D) in the context of obesity and to identify biomarkers with potential for novel treatment strategies. Circulating myeloperoxidase (MPO), piezo-type mechanosensitive ion channel component 1 (PIEZO1), branched-chain amino acids, particularly BCKDH phosphatase, and lectins, such as Gal-4, may serve as inflammatory markers in comorbid obesity and T2D. α-2-Macroglobulin (α2-MG) may be a possible predictive marker in the comorbidity. Angiopoietin-like protein 6 (ANGPTL6) levels may be involved in the T2D pathway. Macrophage and THP-1 exosomes have been shown to be involved in the regulation of inflammation and reduce the expression of proinflammatory genes in mouse models. A thorough understanding of the pathways linking diabetes and obesity is essential for effective prevention, diagnosis, and treatment in T2D patients with obesity. Further research into these biomarkers may contribute to improved patient outcomes and more effective therapeutics.

## 1. Introduction

Diabetes poses a tremendous health burden globally. In 2021, its global prevalence in ages 20–79 was estimated to be 10.5% (536.6 million individuals); this is predicted to rise to 12.2% (783.2 million individuals) by 2045 [1]. It is caused by chronically high levels of blood glucose as a result of the inability of pancreatic β cells to produce adequate amounts of insulin or ineffective insulin utilization by cells [2]. Diabetes can generally be classified as type 1 diabetes (T1D) and type 2 diabetes (T2D), with T2D being the most common. Due to the chronic nature of the disease, diabetes is likely to increase the risk of other diseases caused by macrovascular and microvascular damage and negatively affect organs, such as the brain, kidneys, heart, and eyes [3,4,5]. With diabetes, there is an increased risk of infection and poorer outcomes of infection treatment [6].

Like diabetes, obesity is a chronic metabolic disease that affects multiple organs. Obesity is strongly associated with non-communicable diseases (NCDs), which killed over 17 million people under 70 years old in 2021 [7]. NCDs include cardiovascular disease, cancer, and diabetes [8]. Clinically defined as having a body mass index (BMI) at or exceeding 30 kg/m^2^, in 2022, one in eight people around the world were diagnosed with obesity [9]. According to the World Obesity Atlas, current trends project that 1.1 billion adults (487 million men and 643 million women) will live with obesity by 2030. The risk of developing a high BMI increases with age. In 147 out of 200 countries, the adult lifetime risk of a high BMI in 2022 exceeded 50% [7].

The management of both diabetes and obesity involves GLP-1s, the maintenance of a healthy lifestyle and diet, and participating in consistent physical activity [10,11]. The treatment of diabetes varies depending on whether the patient has T1D or T2D. T1D patients require insulin injections, while those diagnosed with T2D may take metformin, sulfonylureas, or sodium–glucose co-transporter type 2 (SGLT-2) inhibitors to lower blood sugar levels [10,11]. However, data regarding GLP-1s are limited, and medications alone cannot solve the obesity and diabetes epidemic. The global focus should be directed towards structural actions such as healthier food manufacturing and pricing, poverty reduction, resource management, and advocacy to prevent the obesity prevalence from increasing [12].

The molecular bases of diabetic and obesity inflammation are similar, involving a network of interconnected pathways: persistent activation of innate immunity, the elevated production of proinflammatory mediators, and chronic low-grade inflammation, perpetuating metabolic dysfunction. These pathways act on both systemic and tissue-specific levels [13,14]. Proinflammatory cytokines include tumor necrosis factor-alpha (TNF-α), interleukin-6 (IL-6), and interleukin-1 beta (IL-1β). TNF-α is primarily secreted by macrophages and adipocytes and directly impairs insulin signaling, leading to insulin receptor desensitization, peripheral insulin resistance, and inflammation [14,15,16]. IL-6, secreted by adipocytes, immune cells, and muscle tissues, induces insulin resistance in adipose tissue and correlates with the body fat percentage and distribution. It also promotes hepatic glucose production, recruits inflammatory cells, and is associated with increased platelet counts [14,17]. An acute increase in IL-6 may have beneficial metabolic effects, while chronic elevation contributes to insulin resistance and tissue damage [18]. IL-1β is produced via the NLR family pyrin domain-containing 3 (NLRP3) inflammasome pathway. NLRP3 facilitates chronic inflammation, triggered by hyperglycemia [19]. In T1D, IL-1β contributes to the destruction of pancreatic β cells by enhancing autoimmune processes and promoting cell apoptosis. In T2D, it leads to insulin resistance and metabolic inflammation [20].

Obesity and diabetes are also both involved in oxidative damage. Hyperglycemia-induced oxidative stress results in the formation of reactive oxygen species (ROS) through different pathways, such as mitochondrial electron transport chain dysfunction, NADPH oxidase activation, and advanced glycation end-product formation. Oxidative stress disrupts insulin signaling by modifying molecules such as insulin receptor substrate-1 through serine phosphorylation. This impairs glucose uptake in tissues and worsens β-cell dysfunction [21]. In obese patients, endothelin-1 is activated and promotes oxidative damage, leading to increased endothelial cell dysfunction and vasoconstriction [14]. Such oxidative changes result in a vicious cycle of metabolic dysfunction, promoting oxidative stress and exacerbating insulin resistance and inflammation [15].

According to the Institute for Health Metrics in 2024, a high BMI was the second-highest risk factor for T2D [8]. In total, 90% of people with T2D are overweight or obese, and the lifetime risk of developing diabetes in men aged 18 or older increases from 7% to 70% as the BMI increases from less than 18.5 to over 35 kg/m^2^ [22,23]. Similarly, the risk for women increases from 12% to 74% [22]. Patients with obesity and diabetes are 21% more likely to have diabetes-related comorbidity and 42% more likely to be diagnosed with obesity-related comorbidities than patients with diabetes who are not obese. Diabetes-related comorbidities include nephropathy, neuropathy, cardiovascular disease, peripheral vascular disease, and metabolic disease, while obesity-related comorbidities include hypertension, hyperlipidemia, and cardiovascular disease. T2D patients with obesity also have a higher total health cost and resource utilization than T2D patients without obesity [24].

Obesity and diabetes are two different yet interrelated diseases with complex mechanisms and affect millions of people. With the projected rise of diabetes and obesity, there is growing concern about the complications and mortality associated with them. Inflammation in diabetes and obesity may be viewed as a predictive biomarker for their associated complications, emphasizing the value of an inflammation-centric perspective regarding the prevention, diagnosis, and treatment of the complications. Although there have been numerous studies conducted on the two diseases alone, few have been performed on patients with the comorbidity, which could yield new potential biomarkers to target their inflammation mechanisms. The diagnostic and prognostic potential of emerging biomarkers can lead to more effective, personalized treatment approaches that go beyond conventional medicinal and lifestyle targets [13]. The current literature review aims to elucidate the pathogenesis of T2D with obesity and highlight potential biomarkers that may provide beneficial clinical inflammation interventions for comorbid patients.

## 2. Materials and Methods

This narrative review summarizes the current evidence on novel mechanistic details of inflammation in T2D and obesity, as well as potential targets for the treatment of these conditions. A literature search using the PubMed database was performed from 30 June 2026 to 30 September 2026 to determine the relevant literature published between 2016 and 2026. Articles published prior to the past decade were excluded to reflect up-to-date information. The search was intentionally limited to PubMed-indexed, English-language, open-access publications to maintain a feasible scope within the time and resource constraints of the review team, while ensuring that the included literature could be accessed and evaluated in full by readers. This restriction may have resulted in the omission of relevant studies indexed in other databases, published in other languages, or available only through subscription access and is therefore recognized as a limitation of the review. The search terms used were “diabetes complications AND inflammation,” “inflammation AND diabetes AND immune,” “T2D AND inflammation AND treatment,” “diabetes AND obesity,” “biomarker AND diabetes AND obesity,” “anti-inflammatory therapeutics AND diabetes,” “inflammation AND diabetes AND obesity,” and “biomarkers AND DM AND obesity.”

Original research studies, systematic reviews, narrative reviews, meta-analyses, non-randomized comparative studies, randomized controlled trials, and case studies addressing inflammation in comorbid obesity and T2D were eligible for inclusion during the initial search. Surveys, interviews, prospective studies, and articles not directly relevant to the review objectives were excluded. However, systematic reviews, narrative reviews, and meta-analyses not written in the past decade were only used for contextual background information rather than as primary data sources. As this was a narrative rather than a systematic review, the search was not intended to identify every eligible publication, and no formal assessment of study quality or risk of bias was performed.

A total of 22 articles were initially identified, of which 10 were selected for review. Following the initial search, articles were screened to minimize redundancy in topics and study objectives. Selection was based on relevance to the discussion of novel findings related to the mechanisms underlying inflammation in T2D and obesity. The review focused on inflammatory biomarkers investigated in both experimental studies involving mouse models and human participants and relevant review articles. Studies were selected to capture both preclinical and clinical evidence, emphasizing findings that have advanced the current understanding of inflammation-associated biomarkers in obesity and T2D. A particular emphasis was placed on biomarkers because of their potential applications as diagnostic indicators or therapeutic targets. As this was a focused narrative review, only studies that directly investigated the mechanistic role of inflammatory biomarkers in obesity and T2D were included.

We extracted the relevant information from each article, including pertinent findings and limitations (Table 1). Kozak et al.’s narrative review on galectin-4 was chosen as a study of a potential biomarker because of its association with inflammation, as well as its possible role in increasing the obesity risk in diabetic patients. The experimental study by Phu et al. was selected based on its direct investigation of mechanisms linking inflammation, immune cell regulation, and metabolic dysfunction in obesity and insulin resistance. The study was particularly relevant as it covered both in vitro and in vivo approaches to demonstrate the impact of macrophage-derived exosomes on inflammatory macrophage polarization, circulating cytokines, immune cell populations, and inflammatory responses in both adipose tissue and the liver. The study by Cátalan et al. was selected for review as they investigated how T2DM and obesity impact the expression of a specific mechanoreceptor (PIEZO1) and how it relates to inflammation; their findings suggest that PIEZO1 could be a potential biomarker for inflammation that results from T2DM and obesity-induced T2DM. The narrative review by Starling-Soares et al. elucidates the mechanism by which BCAA accumulation occurs through a high-fat diet in diabetic and obese animal models, which may be useful in understanding the role of BCAAs in inflammation in comorbid patients. The randomized controlled study by Consentino et al. was chosen as it analyzed BCAA levels in obese patients and examined how BCAAs may be associated with insulin resistance and increased inflammation. Zhenyukh et al.’s experimental study was selected because it analyzed how elevated BCAA may promote oxidative stress and inflammation in replicated diabetic conditions. Vanweert et al.’s randomized placebo trial and Karusheva et al.’s randomized controlled crossover trial on the dietary restriction of BCAAs were chosen because of its potential to decrease inflammation and insulin resistance in diabetic patients. The study by Wang et al. was chosen as it explored potential inflammatory and metabolic biomarkers associated with T2DM and obesity. The study examined differences in serum protein levels among healthy individuals, individuals with obesity, and individuals with obesity and T2DM, with the findings pointing towards the potential relevance of inflammatory and metabolic proteins in assessing obesity and T2DM.

## 3. Results

### 3.1. Galectin-4 (Gal-4)

Kozak et al.’s literature review, conducted in 2025, analyzed lectins—specifically Gal-4—for individuals with obesity and diabetes. Lectins are proteins that can bind to specific carbohydrate targets to play a variety of roles, such as in cell–cell interactions, signaling pathways, and immune system responses [35]. Gal-4 is expressed in the gastrointestinal tract and is known to stabilize lipid rafts, promote cell cycle progression, and contribute to host cell defense by enhancing the inflammatory response via IL-6 production [25]. Kozak et al. note that elevated Gal-4 concentrations were found in diabetic patients and were associated with a higher likelihood of obesity among diabetic patients with heart failure. Increased Gal-4 also increased the risk of mortality and future cardiovascular events, such as heart disease, heart failure, atherosclerosis, and stroke. Data obtained from studies such as the Malmö Preventive Project suggest that diabetic patients have elevated Gal-4 levels, which increases their risk of obesity [25].

### 3.2. Myeloperoxidase (MPO) and Angiopoietin-like Protein 6 (ANGPTL6)

In a cross-sectional study at the Dasman Diabetes Institute in Kuwait by Qaddoumi et al., 238 adult participants were evaluated to investigate the association of circulating MPO and ANGPTL6 as possible biomarkers for obesity and T2D. MPO is a key inflammatory enzyme used by the immune system; it is stored in white blood cells to kill invading microbes [36]. Previous mouse models found that MPO deficiency conferred resistance to diet-induced obesity and insulin resistance, whereas elevated MPO levels in prediabetic individuals are associated with increased cardiovascular risk [26]. ANGPTL6 is a liver-derived glycoprotein that is involved in upregulating angiogenesis, epidermal cell proliferation, and wound healing. Animal knockout studies have demonstrated that ANGPTL6 enhances energy expenditure, thus counteracting insulin resistance and T2D [26]. To isolate these pathways in humans, patients with T1D, morbid obesity, a history of major chronic illness, current use of medications or supplements altering body/bone composition, or active participation in structured physical exercise within the last 6 months were excluded. The remaining cohort comprised 101 patients with T2D (65 obese, defined as 30  ≤  BMI  <  40 kg/m^2^; 36 non-obese, defined as 19.5 ≤ BMI  <  30 kg/m^2^) and 137 controls without T2D (60 obese; 77 non-obese) (Table 2) [26].

Blood samples were analyzed for fasting plasma glucose, HbA1c, triglycerides, total cholesterol, MPO, and ANGPTL6. The study found that patients with T2D and obesity had overall higher levels of ANGPTL6, and those with T2D had higher levels of ANGPTL6 than those without T2D. It was also found that patients who were obese had higher levels of MPO than non-obese patients both with and without T2D. However, the MPO levels between patients with T2D and those without T2D did not show a statistically significant difference (*p* = 0.26). Finally, higher levels of MPO were associated with higher serum levels of ANGPTL6, triglycerides, TNF-α, hsCRP, IL-6, and PAI-1 and a higher BMI [26].

### 3.3. Macrophage-Derived Exosomes

The study by Phu et al. investigated the function of exosomes produced by THP-1 macrophages and the potential role of M2-like macrophage exosomes in controlling inflammation in the liver and adipose tissue using mouse models. The study focused on human THP-1 macrophages exposed to interleukin-4 (IL-4) as a source of exosomes. In vitro naive THP-1 macrophages and primary bone marrow-derived macrophages (BMDMs) were treated with IL-4-stimulated THP-1 exosomes (THP1-IL4-exo), naive THP1 exosomes (THP1-WT-exo), PBS (control), or direct IL-4 treatment (positive control). The result showed that THP1-IL4 exosomes promoted anti-inflammatory macrophage polarization in a dose-dependent manner. At an exosome concentration of 2 × 109 particles/mL, there was reduced expression of M1 genes, such as Tnf, Il1b, Cd86, and Cd80, and increased expression of M2 markers, such as Cd206 and Cd163. At 4 × 109 particles/mL, the effects examined were stronger, and the gene expression changes produced were similar to those under direct treatment with IL-4. THP1-WT-exo demonstrated a reduction in proinflammatory gene expression, but this was two-to-three-fold smaller than for THP1-IL4-exo. Furthermore, exosomes from THP-1 macrophages treated with 100 ng/mL LPS and 20 ng/mL IFN-γ showed the upregulated expression of Tnf, Il1b, Cd86, and Cd80. PKH26 fluorescent labeling showed that BMDMs readily took up THP1-IL4 exosomes. The internalization efficiency was similar to that observed in THP-1 macrophages. Following uptake, BMDMs also displayed increased anti-inflammatory gene expression [27].

Phu et al. also treated obese, insulin-resistant mice with a diet rich in saturated fat and sucrose and with THP1-IL4 exosomes derived from IL-4-exposed BMDMs. Both THP1-WT-exo and THP1-IL4-exo showed similar distributions in multiple organs: epididymal white adipose tissue (eWAT), lung, liver, brain, kidney, aorta, spleen, bone, and intestine. Despite repeated treatment, body weight and fat mass remained unchanged. Treatment with THP1-IL4 exosomes led to reductions in the amounts of circulating CD45+ CD11b+ myeloid cells, inflammatory Ly6Chi monocytes, neutrophils, and inflammatory monocytes and neutrophils in the spleen. There was also a reduction in the abundance of several hematopoietic stem and progenitor cells in the bone marrow and spleen, including Lin− Sca-1+ c-Kit+ (LSK) cells, multipotent progenitors (MPP1–4), common myeloid progenitors (CMPs), granulocyte–macrophage progenitors (GMPs), and megakaryocyte–erythroid progenitors (MEPs). Exosomes reduced the expression of proinflammatory genes (Tnf, Il1b, and Mcp1) and increased the expression of genes involved in lipid metabolism and anti-inflammatory function (Pparg, Abca1, and Abcg1). They also reduced the expression of miR-33-5p, as well as Srebf1 and Srebf2, which are involved in cholesterol and lipid synthesis regulation. Lastly, THP1-IL4-exo increased several anti-inflammatory microRNAs in inflammatory monocytes, such as miR-99a-5p, miR-146b-5p, miR-378a-3p, and miR-21-5p. Treatment with THP1-IL-exo resulted in lower circulating levels of inflammatory cytokines, such as TNF-α, IFN-γ, IL-6, and IL-1β. Repeated administration of THP1-IL4-exo significantly reduced leukocyte infiltration into the aorta, liver, and adipose tissue and showed reductions in inflammatory macrophages, Ly6Chigh monocytes, and neutrophils. The exosomes also shifted tissue macrophages towards the M2-like anti-inflammatory phenotype. These effects were similar in both genetically hyperlipidemic and Western diet-fed wild-type obese mice [27].

### 3.4. Piezo-Type Mechanosensitive Ion Channel Component 1 (PIEZO1)

**Figure 1:**
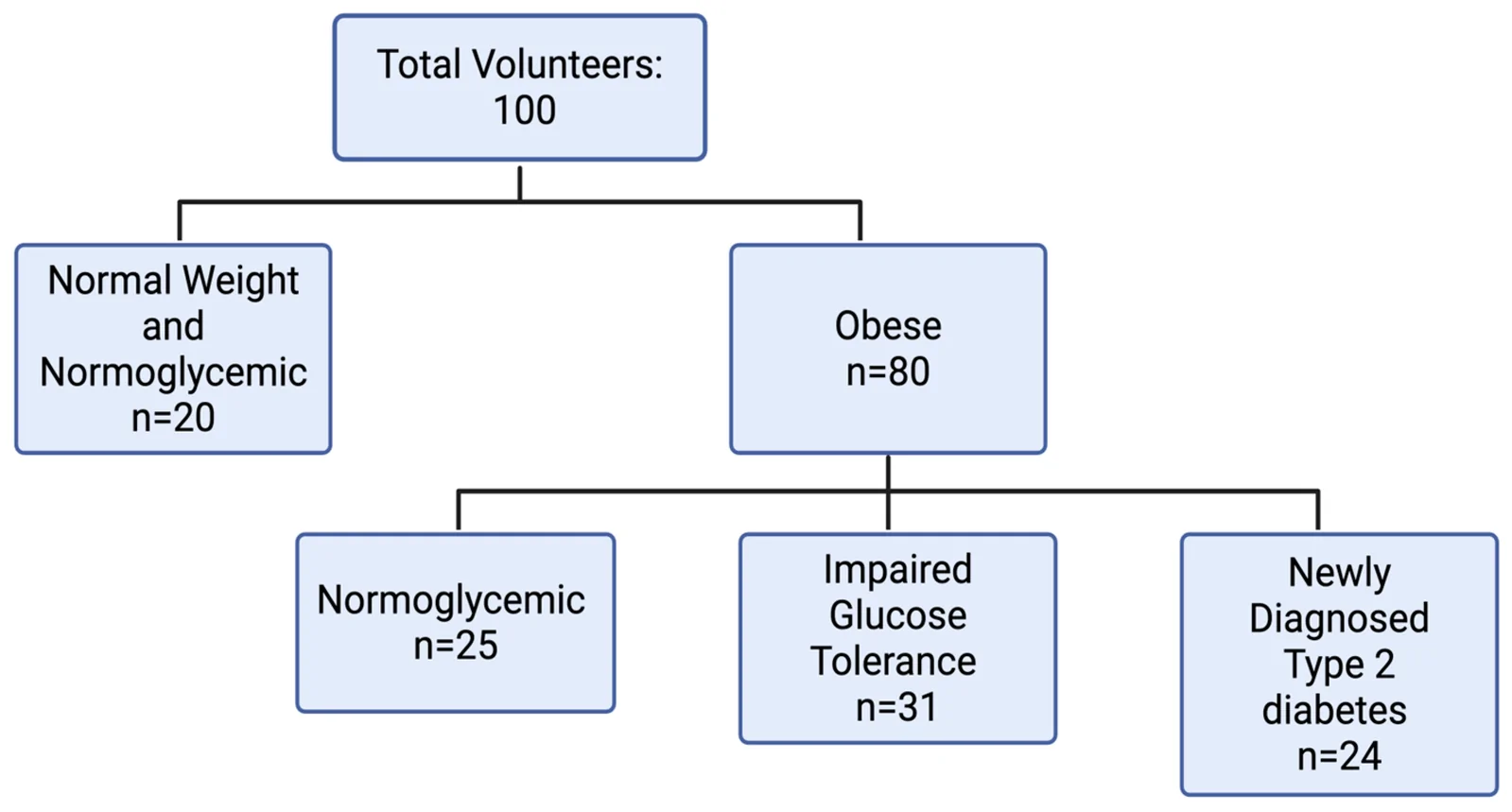
Grouping and sample sizes of volunteers in Cátalan et al.’s study. A total of 100 volunteers were categorized as either normal weight or obese. Obese volunteers were further categorized based on their glycemic status [28]. Created in BioRender. Han, J. (https://BioRender.com/oa4q8ip, accessed on 11 September 2026) is licensed under CC BY 4.0.

An experimental study by Catálan et al. investigated PIEZO1 levels within adipose tissue in individuals with obesity and T2D. PIEZO1 is a cation channel on plasma membranes that responds to mechanical stimuli. PIEZO1 acts in conjunction with other depolarizing channels, including SWELL1, an anion channel that is volume-regulated [28]. In a study conducted in 2021, PIEZO1 was linked to inflammation; tissue stiffness was associated with increased expression of PIEZO1 and Ca2+ activity, leading to inflammation through an increase in inflammatory markers such as interferon-*γ* and lipopolysaccharide (LPS) [37]. Catálan et al. proposed that the functioning of PIEZO1 was dysregulated in individuals with obesity and individuals with obesity associated with T2D, leading to the inflammation of adipose tissue. To investigate this hypothesis, they conducted a study on 100 volunteers. Of the 100 volunteers, 59 were female and 41 were male. Twenty volunteers were of a normal weight and normoglycemic; 80 volunteers were diagnosed with obesity. The volunteers with obesity were further divided into three groups as follows; those who were normoglycemic, those with impaired glucose tolerance, and those who were newly diagnosed with T2D. Individuals with T2D were not on medications that affected their insulin levels during the study (Figure 1) [28]. Biopsies of visceral adipose tissue and serum samples showed that, in individuals with obesity and obesity with T2D, there were increased mRNA levels of PIEZO1 (*p* < 0.05). There was a significant association between PIEZO1 and BMI (*p* = 0.002), weight (*p* = 0.010), and body fat (*p* = 0.024) [28]. Additionally, the mRNA levels of PIEZO1 and SWELL1 were positively associated, and the mRNA levels of the SWELL1 receptor were increased in those with obesity who were normoglycemic (*p* < 0.05) and those with obesity and T2D (*p* < 0.001). A positive correlation was found between PIEZO1 and all inflammation markers. Specifically, the mRNA levels of PIEZO1 and SWELL1 were associated with those of IL-18 and IL-1B (*p* < 0.05) after controlling for body weight and body fat. Finally, Catalán et al. also found that the compression of visceral adipose tissue led to increased expression of PIEZO1, resulting in an inflammatory response [28].

### 3.5. Branched-Chain Amino Acids (BCAAs)

BCAAs include amino acids, such as leucine, valine, and isoleucine, which accumulate during excess food intake. A narrative review conducted by Starling-Soares et al. proposed that a high-fat diet (HFD) in diabetic and obese animal models induced a decrease in branched-chain ketoacid dehydrogenase (BCKDH) phosphatase in the liver tissue, white adipose tissue, and peritoneal macrophages, which then increased branched-chain ketoacid kinase (BCKDK) activity. This increased kinase activity decreases BCKDH utility, preventing BCAA degradation, further causing BCAA accumulation in the body [29].

In a randomized controlled study by Consentino et al., 15 obese youth (ages 14–18 years) were compared with six normal-weight youth participants. Obese youth were further split into an intervention group, who were asked to perform physical activity while monitored, and control groups, who were given lifestyle advice but were not actively monitored. Blood samples were used to measure a variety of concentrations, such as BCAA, insulin, plasma glucose, and IL-6. The total BCAA concentration was significantly higher in obese patients than normal-weight patients (*p* = 0.004) and had a positive association with IL-6 (r = 0.52, *p* = 0.01), the log of high-sensitivity C-reactive protein (r = 0.53, *p* = 0.01), and Homeostatic Model Assessment for Insulin Resistance (HOMA-IR) (r = 0.45, *p* = 0.04) values. There were no significant findings for the intervention group [30].

**Figure 2:**
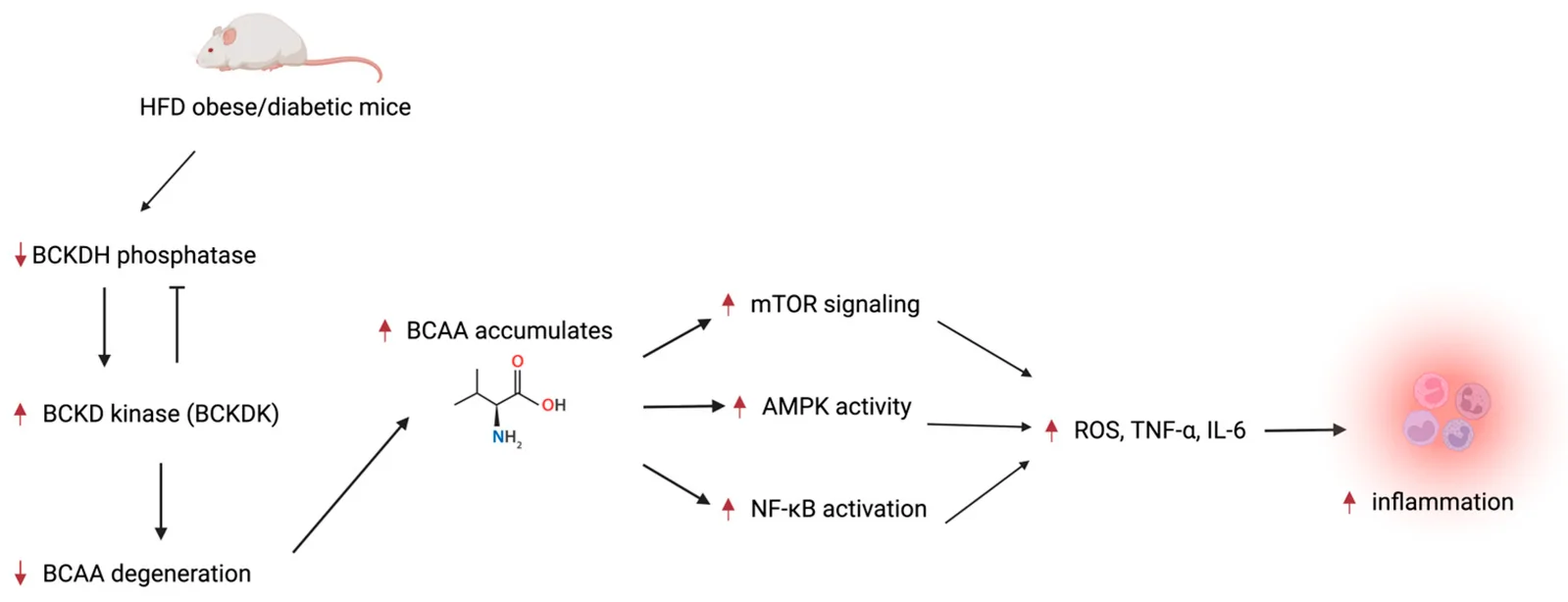
Mechanism of BCAA-induced inflammation in comorbidity. A high-fat diet decreases BCKDH, which causes BCAA accumulation. High levels of BCAAs upregulate and activate signaling pathways that increase inflammatory cytokines, causing inflammation [29,31]. Created in BioRender. Han, J. (https://BioRender.com/98phrde, accessed on 11 September 2026) is licensed under CC BY 4.0.

In addition, Zhenyukh et al. found that peripheral blood mononuclear cells (PBMCs) and elevated BCAAs promoted oxidative stress and inflammation. As the BCAA concentrations increased, NADPH oxidase activity increased (*p* = 0.05). To replicate the hyperglycemic conditions found in diabetics, PBMCs were exposed to high glucose levels. In this state, BCAAs at a lower concentration enhanced NADPH oxidase activity (*p* = 0.05). BCAAs were also found to significantly induce mTOR signaling, upregulate AMP-activated protein kinase (AMPK) activity, and activate the NF-κB pathway. Through these induced pathways, ROS production (*p* = 0.05) and TNF-α (*p* = 0.01) and IL-6 levels were increased (*p* = 0.01) (Figure 2) [31].

To test BCAA diet restriction, patients with well-controlled T2D were given sodium phenylbutyrate (NaPB), a branched-chain α-keto acid dehydrogenase complex (BCKD) kinase inhibitor, to decrease their BCAA levels. A total of 16 patients participated in the study, with eight receiving NaPB treatment and eight others receiving placebos. After two weeks of treatment, metabolism, substrate oxidation, muscle biopsy, and two-step euglycemic hyperinsulinemic clamp measurements were performed. The NaPB treatment group had improved whole-body insulin sensitivity compared to the placebo group (*p* = 0.02), as well as enhanced muscle mitochondrial oxidative capacity (*p* = 0.04) [32]. Another study analyzed BCAA diet restriction in overweight/obese (BMI 28–35 kg/m^2^) and T2D patients. The 12 patients that participated were given either a powder that contained all amino acids or a powder lacking BCAAs. BCAA-restricted patients showed increased insulin release (*p* < 0.05), lower C-peptide release (*p* < 0.05), and decreased mTOR levels compared to controls (*p* < 0.05) [33].

### 3.6. Proteomic Biomarkers

At Inner Mongolia Medical University, Wang et al. recruited a total of 229 males (ages 40–55). Among these, 73 were categorized as the healthy control group, 80 were categorized as having obesity (BMI > 30 kg/m^2^) alone, and 76 were categorized as having newly diagnosed T2DM (initial fasting plasma glucose ≥ 7.0 mmol/L) and obesity. After the participants’ blood samples were collected, they were analyzed via two-dimensional gel electrophoresis (2-DE), enzyme-linked immunosorbent assay (ELISA), and electrospray ionization quadrupole time-of-flight tandem mass spectrometry (ESI-Q-TOF-LC/MS) to assess changes in serum protein (Table 3).

**Table 3:** Summary of protein serum levels among control, obesity, and T2DM + obesity groups in the study by Wang et al. The average α-2-macroglobulin (α2-MG), zinc-α2-glycoprotein (ZAG), complement component C3 (C3), and α1-antitrypsin (AAT) serum levels are summarized below to demonstrate the differences among the 3 groups. Created in BioRender. Nguyen, J. (https://BioRender.com/qiilyx2, accessed on 11 September 2026) is licensed under CC BY 4.0.

		Control Group	Obesity Group	T2DM + Obesity Group
**Protein Serum Levels**	**α2-MG** (g/L)	1.376 ± 0.229	2.746 ± 0.391	3.261 ± 0.400
	**ZAG** (μg/mL)	57.578 ± 25.767	47.968 ± 31.916	44.303 ± 23.630
	**C3** (g/L)	1.076 ± 0.303	1.201 ± 0.302	1.565 ± 0.296
	**AAT** (g/dL)	3.086 ± 0.596	2.753 ± 0.454	2.539 ± 0.516

Based on gel scan analyses, the control group had a mean of 113 ± 33 protein spots, the group with obesity had a mean of 126 ± 39, and the group with T2DM + obesity had a mean of 123 ± 27. Among the three groups, eight protein spots were significantly expressed at various concentrations (*p* < 0.01): complement C4-A, clusterin, α-2-macroglobulin (α2-MG), zinc-α2-glycoprotein (ZAG), complement C3 (C3), apolipoprotein L1, α1-antitrypsin (AAT), and complement factor B. Using proteomic analysis and ELISA, the concentrations of the proteins C3 (*p* = 0.000) and α2-MG (*p* = 0.000) were discovered to be underexpressed in the control group, while AAT (*p* = 0.000) and ZAG (*p* = 0.011) were found to be overexpressed. The C3 levels were 1.565 ± 0.296 g/L in the T2DM + obesity group, 1.201 ± 0.302 g/L in the obesity group, and 1.076 ± 0.303 g/L in the control group. The α2-MG levels were 3.261 ± 0.400 g/L in the T2DM + obesity group, 2.746 ± 0.391 g/L in the obesity group, and 1.376 ± 0.229 g/L in the control group. The ZAG levels were 57.578 ± 25.767 μg/mL in the control group, 47.968 ± 31.916 μg/mL in the obesity group, and 44.303 ± 23.630 μg/mL in the T2DM + obesity group. The AAT levels were 3.086 ± 0.596 g/dL in the control group, 2.753 ± 0.454 g/dL in the obesity group, and 2.539 ± 0.516 g/dL in the T2DM + obesity group [34].

A multivariate logistic regression analysis revealed that the increased levels of C3 (OR = 3.580, *p* < 0.01) alongside α2-MG (OR = 11.537, *p* < 0.01) found in the obesity group were significant contributors to the development of obesity when compared to the control participants. Likewise, the reduced AAT levels (OR = 0.997, *p* < 0.05) and increased C3 (OR = 7.202, *p* < 0.01) and α2-MG levels (OR = 16.801, *p* < 0.01) of the T2DM + obesity group were a significant factor in the development of T2DM. While the receiver operating characteristic curve analysis concluded that the proteins C3, ZAG, and α2-MG were all significant predictors of obesity, α2-MG had the highest sensitivity (AUC = 0.873). C3 and ZAG were the only sensitive and significant predictors in diagnosing obesity alone. AAT (AUC = 0.646) and α2-MG (AUC = 0.601) levels were significant predictors of T2DM + obesity [34].

## 4. Discussion

The interactions between obesity and diabetes are complex and often not well-studied. The mechanisms and pathways by which elevated Gal-4 concentrations are associated with obesity, diabetes, and cardiovascular disease are not yet known. According to Kozak et al., only the Malmö Preventative Project has so far conducted studies on the association of Gal-4 and obesity in patients; these took place in 2022 and 2023 [25]. More studies are necessary to better understand this association and determine whether Gal-4 can be a marker for diabetes, obesity, or both. The investigation of elevated Gal-4 levels and their association with an increased risk of cardiovascular disease also may be useful for patients with diabetes and obesity. Since the role and utility of Gal-4 are still emerging, more research should be conducted on its mechanisms associated with heart failure.

The findings of Qaddoumi et al. showed the possibility of a complex interconnection between the immunometabolic system via biomarkers MPO and ANGPTL6. The evaluation of these two biomarkers in tandem helped to further show an association with T2D and obesity in humans, whereas they have previously been seen in animal and mouse models. However, the translation of these biomarkers into clinical application will require a deeper understanding of and further research into their specific mechanisms, roles, and viability as drug targets. The paradoxical finding that ANGPTL6, which was found in animal models to increase insulin sensitivity, was elevated in patients with T2DM and obesity may indicate a critical point in the metabolic feedback loop, namely compensatory hypersecretion as a downstream reaction to hyperinsulinemia. On the other hand, Qaddoumi et al. found that MPO was elevated in obese patients regardless of diabetic status and had a strong positive association with inflammatory cytokines, indicating MPO as part of the neutrophil-mediated oxidative stress driver and its relation to systemic vascular damage. As current models of diagnostics for obesity and prediabetes rely on glycemic markers, they do not accurately account for tissue pathology. The possibility of using ANGPTL6 and MPO as dual biomarkers in panels may aid in the diagnosis of early-stage adipose inflammation. The use of the MPO and ANGPTL6 pathways may also be a viable option for novel drug targeting. However, further study of their mechanisms is required to accurately determine whether ANGPTL6 and MPO are viable targets, seeking to understand whether they precede or are downstream products of T2D development [26].

The study by Phu et al. built upon previous findings that M1-like macrophages in the liver, adipose tissue, and vascular tissue are proinflammatory and contribute to chronic inflammation, leading to cardiometabolic disease and its complications [38]. The study did not involve human patients or subjects; it relied on human THP-1 cells for the exosome-producing cell line, mouse BMDMs and 3T3-L1 cells for some in vitro experiments, and mice for the in vivo experiments. The researchers previously found that exosomes derived from mouse BMDMs cultured with elevated glucose levels resulted in exosomes with altered microRNA cargo, including high levels of miR-486-5p. This microRNA downregulates the expression of Abca1 in macrophages. These macrophage exosomes were found to drive hematopoiesis and accelerate atherosclerosis when infused into Apoe^−/−^ mice [39]. Such findings support the notion that exosomes produced by macrophages exposed to inflammatory cytokines and metabolic stress are contributors to cardiometabolic disease. However, new findings have illustrated that exosomes produced by alternatively activated M2-like macrophages could contribute to inflammation control. The researchers confirmed this new claim with M2-like BMDMs exposed to IL-4-producing exosomes in Apoe^−/−^ mice [40]. Further study would be necessary to establish this relationship in humans.

By studying both human THP-1 macrophages and primary mouse BMDMs, Phu et al. showed that the exosome effects are not limited to THP-1 cells but also occur in primary macrophages [27]. BMDMs provided supporting evidence that the effects were observed in human THP-1 macrophages and primary mouse macrophages. This allowed more detailed metabolic studies, strengthening the findings. Since IL-4 exosomes mimicked the result of direct IL-4 treatment, this suggests that IL-4 conditioned exosomes can transfer many of the biological effects of IL-4 to recipient macrophages. THP1-IL4-exo can act as potent anti-inflammatory signaling vesicles; they are internalized by recipient macrophages, suppress proinflammatory (M1) gene expression, and promote anti-inflammatory (M2) polarization. These findings suggest that the exosomes transmit immune and metabolic reprogramming signals, reflecting the activation state of the parent cells. The study has revealed that the exosomes are sufficient to transfer many phenotypic features of the parent cells, reinforcing the immune environment established. However, this does not prove that the exosomes are the main mechanism by which macrophages coordinate tissue responses. In contrast to the in vitro setting of this study, soluble cytokines, chemokines, cell-to-cell contact, and exosomes all work together in vivo.

With both THP1-WT-exo and THP1-IL4-exo showing similar biodistribution, the differences in their biological effects were likely due to their cargo rather than where they traveled in the mice. In the obese mouse models, the exosomes did not affect obesity itself as there were no changes in body weight or fat mass; the reduction in inflammation occurred without causing weight loss [27]. Given this information, the observed immunological effects can be seen as weight-independent. This strengthens the conclusion that the exosomes directly modulated immune cell production and inflammatory responses, rather than indirectly improving inflammation by making the mice leaner. From a therapeutic perspective, these preclinical findings suggest that THP1-IL4 exosomes may be able to reduce obesity-associated inflammation without the need for weight loss. If sustained weight loss is difficult to achieve, a treatment that reduces inflammatory disease independently of weight reduction could have substantial clinical value. However, this study alone does not reflect the long-term outcomes of exosome treatment in human subjects.

In the mouse models, through the suppression of hematopoiesis, THP1-IL4-exo decreased the production of inflammatory myeloid cells at their source. The changes in the levels of expression of regulatory miRNAs likely contributed to the observed changes in inflammatory and metabolic gene expression. Decreased levels of circulating inflammatory cytokines demonstrated that THP1-IL4-exo were able to achieve a reduction in systemic inflammatory signaling [27]. The study by Phu et al. supports a model in which IL-4-conditioned macrophage-derived exosomes act as systemic immunometabolic regulators. In the treated mice, the exosomes reprogrammed circulating immune cells and hematopoiesis but also reduced inflammatory cell infiltration and promoted tissue repair across multiple organs, including blood vessels, the liver, and adipose tissue. This ability suggests their potential therapeutic value for obesity-related cardiometabolic disease. The study did measure metabolic outcomes relevant to diabetes in mice, including fasting blood glucose, insulin levels, and glucose tolerance. THP1-IL4-exo treatment improved these parameters in obese mice. However, these findings have not been established in human subjects.

Obesity and T2D can lead to changes within the microenvironment of adipose tissue, ultimately causing inflammatory responses. As visceral white adipose tissue accumulates in the body, the adipocytes within the tissue begin to shrink and expand as needed. The adipocytes are supported by the extracellular matrix (ECM), which is constantly remodeling to support and regulate the changing environment. An increase in ECM remodeling is a natural, beneficial response to temporary instances of excess caloric intake. However, in chronic obesity, the ECM loses the ability to remodel appropriately, leading to abnormal organization associated with inflammation and adipose tissue dysfunction [41].

The study by Catálan et al. provides valuable insight into these concepts by identifying a correlation between PIEZO1 and inflammation. PIEZO1 expression increases with the compression of adipose tissue, and PIEZO1 is positively correlated with inflammation [28]. Thus, the higher the level of expression of PIEZO1, the more inflammation can occur. With this knowledge, PIEZO1 has the potential to serve as a biomarker for inflammation resulting from obesity or obesity associated with T2D. Although more research is needed to understand the complete influence of PIEZO1 within adipose tissue in obesity, this knowledge can be used to conduct further research and testing to assess whether PIEZO1 has potential as a target for weight loss or weight control medications.

Through a complex network of cellular signaling and pathways, the accumulation of BCAAs from excess food intake increases oxidative stress, inflammation, and insulin resistance in obese and diabetic patients. Diabetic patients are especially at risk from inflammation since NADPH oxidase is enhanced in high-glucose conditions [31]. As discussed before, this consistent source of inflammation and oxidative stress may worsen β dysfunction and contribute to insulin resistance and impaired glucose uptake. Because of BCAAs’ extensive role in inflammatory pathways, restricting BCAAs in the diet may be a potential therapeutic strategy for comorbid patients with T2D and obesity. By decreasing BCAA levels in comorbid patients, it may be possible to prevent the overactivation of inflammatory pathways such as mTOR, as well as lowering insulin resistance, which may improve the T2DM prognosis. However, within the few human studies available regarding BCAA diet restriction, the sample size was minimal, with no more than 20 participants [32,33]. The time allocated for both trials was also short, highlighting the need for a longer study with a greater sample size before BCAA restriction can be utilized as a potential therapeutic strategy for comorbid patients with T2DM and obesity.

Although elevated BCAAs show promise as a biomarker for increased inflammation in comorbid patients, information regarding BCAAs’ role in inflammation is controversial. Starling-Soares et al. identified studies from Bugajska et al. and Katagiri et al. that show no correlation between BCAA, TNF-α, and IL-6 levels, while other research, such as the studies conducted by Consentino et al. and Zhenyukh et al., contradicts these findings [29,30,31]. Consentino et al.’s study had a small sample size focusing on youth, which may not be representative of the general population [30]. A study with a larger sample size that includes adults with varying demographic backgrounds may increase the strength of Consentino et al.’s findings. Similarly, Zhenyukh et al. conducted a study on PBMC cultures, rather than collecting samples from patients [31]. These pathways may interact with BCAAs differently while still in the human body, especially in those who are obese or diabetic. The population from which the PBMCs were obtained was undisclosed. A similar study that replicates Zhenyukh et al.’s should be conducted on obese and diabetic patients to validate their findings.

The study by Wang et al. concluded that AAT serum levels demonstrated high specificity for distinguishing participants with T2DM; serum C3 and ZAG levels had limited predictive value; and α2-MG serum levels had high sensitivity and specificity for predicting both obesity and T2DM in the study population [34]. Due to the sample size, there are some limitations in the representativeness of the results regarding the broader population. The reported results reflect the performance of the biomarkers within the population and study design used by Wang et al.; therefore, larger studies involving diverse populations are needed to determine whether α2-MG consistently serves as a predictive factor for diabetes. α2-MG is a large glycoprotein produced by the macrophages of the liver through the activation of transcription factors [42]. This glycoprotein has the ability to inhibit proteases and regulate the activity of cytokines, hormones, growth factors, and other proteins [43]. This could be due to the fact that the body secretes protease inhibitors, such as α2-MG, during the prediabetic stage of insulin resistance in obesity. α2-MG alleviates insulin resistance by binding to low-density lipoprotein receptor-related protein-1, which promotes the expression of glucose transporter type 4 on the cell surface [44,45]. The obese participants in the study had significantly higher levels of serum α2-MG compared to the control group before attaining the glycemic diagnostic threshold for DM. Further investigation will help to clarify whether this biomarker could be used as an early warning sign for the progression from insulin resistance to diabetes [34].

Since the data extracted from this narrative review were limited, it used a single database, it did not include a formal quality assessment, and it largely drew from cross-sectional, animal, or preclinical studies, the potential clinical use of these candidate biomarkers should not be considered until further evidence of their efficacy and therapeutic benefits is obtained.

## 5. Conclusions

Obesity and diabetes increase the risk of serious complications and mortality, highlighting the need for early prevention and intervention. The investigation of macrophage-derived exosomes in human studies and further studies of biomarkers such as Gal-4, MPO, ANGPTL6, BMDM, PIEZO1, BCAAs/BCKDH phosphatase, and α2-MG may clarify the inflammatory mechanisms underlying T2D and obesity and lead to improved diagnostic, disease monitoring, and therapeutic strategies.

## Figures and Tables

**Table 1:** Summary of selected studies on molecular mechanisms linking obesity and type 2 diabetes: pathogenesis, biomarkers, and therapeutic potential. Overview of included publications, stratified by author, study design, population, biomarker evaluated, main results, and main limitations of each study.

Author	Study Design	Population (N)	Biomarker Evaluated	Main Result	Main Limitations
Kozak et al. (2025) [25]	Narrative literature review	N/A	Galactin-4 (Gal-4)	Elevated Gal-4 in diabetic patients is associated with an increased risk of obesity.	Molecular mechanism linking Gal-4 to obesity remains unclear.
Qaddoumi et al. (2020) [26]	Cross-sectional observational study	N = 238	Myeloperoxidase (MPO) and angiopoietin-like protein 6 (ANGPTL6)	ANGPTL6 levels were elevated in patients with T2D and obesity. MPO was significantly higher in obese individuals regardless of diabetic status.	Paradoxical elevation of ANGPTL6 in human T2D/obesity indicates a complex compensatory response.
Phu et al. (2022) [27]	Experimental preclinical study	N/A	Macrophage-derived exosomes (IL-4-stimulated THP-1 exosomes/THP1-IL4-exo)	THP1-IL4 exosomes promoted anti-inflammatory M2 macrophage polarization, suppressed M1 proinflammatory cytokines, inhibited bone marrow hematopoiesis, and improved insulin sensitivity in obese mice.	Preclinical study lacking human clinical trial evaluation.
Catálan et al. (2024) [28]	Experimental/cross-sectional human study	N = 100	Piezo-type mechanosensitive ion channel component 1 (PIEZO1)	PIEZO1 mRNA levels in visceral adipose tissue were significantly increased in obesity and obesity with T2D.	Exact mechanotransduction signaling cascades and the clinical safety of targeting PIEZO1 require further investigation.
Starling-Soares et al. (2026) [29]	Narrative literature review	N/A	Branched-chain amino acids (BCAAs)	Accumulated BCAAs from increasing BCKDK due to high-fat diet trigger mTOR, AMPK, and NF-κB signaling, elevating ROS and inflammatory cytokines.	Human literature regarding BCAAs’ correlation with TNF-α and IL-6 remains conflicting across studies.
Consentino et al. (2021) [30]	Randomized controlled intervention study	N = 21	Branched-chain amino acids (BCAAs)	BCAA levels positively correlated with inflammatory cytokines (IL-6, hsCRP) and insulin resistance in obese youth.	Small sample size that was restricted strictly to adolescents.
Zhenyukh et al. (2017) [31]	In vitro experimental cell study	N/A	Branched-chain amino acids (BCAAs)	Elevated BCAAs stimulated NADPH oxidase activity, ROS production, mTOR signaling, AMPK activity, and NF-κB pathway, increasing TNF-α and IL-6 release and cell migration.	In vitro cell culture model lacks systemic, tissue-level, and metabolic interactions present in human organisms.
Vanweert et al. (2022) [32]	Double-blind, randomized, placebo-controlled crossover trial	N = 16	Branched-chain amino acids (BCAAs)	Two weeks of NaPB treatment reduced plasma [BCAAs], which improved peripheral insulin sensitivity and enhanced muscle mitochondrial oxidative capacity.	Small sample size and short treatment timeframe, lacking assessment of long-term durability and safety of chronic NaPB administration.
Karusheva et al. (2019) [33]	Double-blind, randomized, controlled crossover trial	N = 12	Branched-chain amino acids (BCAAs)	Reduction in BCAA levels decreased meal-induced insulin secretion, increased oral glucose sensitivity, and increased FGF1 levels.	Small sample size and short treatment duration.
Wang et al. (2025) [34]	Cross-sectional proteomic comparative study	N = 229	α-2-macroglobulin (α2-MG), complement component C3 (C3), zinc-α2-glycoprotein (ZAG), and α1-antitrypsin (AAT)	α2-MG and C3 serum levels were significantly elevated in obesity and T2DM + obesity groups compared to controls. AAT was decreased in T2DM + obesity.	Study cohort was strictly limited to adult males aged 40–55 years from a single center, limiting generalizability to females, different age groups, and broader populations.

**Table 2:** Cross-sectional cohort stratification by type 2 diabetes status and obesity classification in the study cohort evaluated by Qaddoumi et al. (N = 238). Obese status was defined as 30 ≤ BMI < 40 kg/m^2^, and non-obese status was defined as 19.5 ≤ BMI < 30 kg/m^2^ [26].

	Obese	Non-Obese	Total
With T2D	65	36	101
Without T2D	60	77	137
Total	125	113	238

## Data Availability

No new data were created in the production of this literature review. The data presented in this review were derived from the following public domain: https://pubmed.ncbi.nlm.nih.gov/.

## Abbreviations

The following abbreviations are used in this manuscript:

α2-MG	α-2-Macroglobulin
AAT	α1-Antitrypsin
AMPK	AMP-activated protein kinase
ANGPTL6	Angiopoietin-like protein 6
BMI	Body mass index
BMDM	Bone marrow-derived macrophage
BCAA	Branched-chain amino acid
BCKDH	Branched-chain ketoacid dehydrogenase
BCKD	Branched-chain α-keto acid dehydrogenase complex
BCKDK	Branched-chain ketoacid kinase
C3	Complement component 3
CMP	Common myeloid progenitor
ELISA	Enzyme-linked immunosorbent assay
ESI-Q-TOF-LC/MS	Electrospray ionization quadrupole time-of-flight tandem mass spectrometry
Gal-4	Galectin-4
GLP-1	Glucagon-like peptide-1
HFD	High-fat diet
IL-4	Interleukin-4
IL-6	Interleukin-6
IL-1β	Interleukin-1 beta
LPS	Lipopolysaccharide
LSK	Lin-Sca-1+ c-Kit+
MEP	Megakaryocyte–erythroid progenitor
MPO	Myeloperoxidase
MPP1–4	Multipotent progenitor cells 1 through 4
NLRP3	NOD, LRR, and pyrin domain-containing protein 3
NCD	Non-communicable disease
PBMC	Peripheral blood mononuclear cell
PIEZO1	Piezo-type mechanosensitive ion channel component 1
ROS	Reactive oxygen species
SGLT-2	Sodium–glucose co-transporter type 2
NaPB	Sodium phenylbutyrate
THP1-IL4-exo	IL-4-stimulated THP-1 exosomes
THP1-WT-exo	Naïve THP-1 exosomes
TNF-α	Tumor necrosis factor-alpha
T1D	Type 1 diabetes mellitus
T2D/T2DM	Type 2 diabetes mellitus
ZAG	Zinc-α2-glycoprotein
